# A detailed staging scheme for late larval development in *Strongylocentrotus purpuratus* focused on readily-visible juvenile structures within the rudiment

**DOI:** 10.1186/1471-213X-14-22

**Published:** 2014-05-19

**Authors:** Andreas Heyland, Jason Hodin

**Affiliations:** 1 University of Guelph, 50 Stone Road East, Guelph, ON N1G 2W1, Canada; 2 Hopkins Marine Station of Stanford University, Pacific Grove, CA 93950, USA

**Keywords:** Sea urchin, Echinoidea, Echinodermata, Skeletogenesis, Rudiment, Metamorphosis, Morphogenesis, Growth, Development

## Abstract

**Background:**

The purple sea urchin, *Strongylocentrotus purpuratus*, has long been the focus of developmental and ecological studies, and its recently-sequenced genome has spawned a diversity of functional genomics approaches. *S. purpuratus* has an indirect developmental mode with a pluteus larva that transforms after 1–3 months in the plankton into a juvenile urchin. Compared to insects and frogs, mechanisms underlying the correspondingly dramatic metamorphosis in sea urchins remain poorly understood. In order to take advantage of modern techniques to further our understanding of juvenile morphogenesis, organ formation, metamorphosis and the evolution of the pentameral sea urchin body plan, it is critical to assess developmental progression and rate during the late larval phase. This requires a staging scheme that describes developmental landmarks that can quickly and consistently be used to identify the stage of individual living larvae, and can be tracked during the final two weeks of larval development, as the juvenile is forming.

**Results:**

Notable structures that are easily observable in developing urchin larvae are the developing spines, test and tube feet within the juvenile rudiment that constitute much of the oral portion of the adult body plan. Here we present a detailed staging scheme of rudiment development in the purple urchin using soft structures of the rudiment and the primordia of these juvenile skeletal elements. We provide evidence that this scheme is robust and applicable across a range of temperature and feeding regimes.

**Conclusions:**

Our proposed staging scheme provides both a useful method to study late larval development in the purple urchin, and a framework for developing similar staging schemes across echinoderms. Such efforts will have a high impact on evolutionary developmental studies and larval ecology, and facilitate research on this important deuterostome group.

## Background

The purple sea urchin, *Strongylocentrotus purpuratus*, has been the focus of developmental and ecological studies for over a hundred years. Its widespread distribution, large population sizes, active fishery, dramatic high energy habitat, ease of obtaining adults and gametes and hardiness as a laboratory study organism has contributed to this long term usage [1]. More recently, *S. purpuratus* was the first free-living, non-chordate marine invertebrate with a fully sequenced genome [2], and the application of modern gene manipulation techniques to the early sea urchin embryo has reinvigorated its use in understanding the basic mechanisms of cell biology and development.

Purple urchins develop through a feeding larval stage that, depending on latitude and food supply [3], spends 1–3 months feeding on phytoplankton before settling to the sea floor and completing metamorphosis into a feeding juvenile. This complex life history determines the nature of dispersal from parental sites and recruitment patterns of juveniles into adult populations [4]. Moreover, the transformation from a larva to a juvenile, in which the bilaterally symmetric larva transforms into the pentameral juvenile urchin, is a fascinating ontogenetic event unto itself.

In contrast to insects and amphibians, relatively little is known about the mechanisms of echinoderm metamorphosis in general, and purple urchin metamorphosis specifically (for what is known see [5-16]). Comparative morphology and functional studies in a phylogenetic context suggest that echinoderm metamorphosis evolved independently from that in insects and amphibians, and that many other marine invertebrate groups themselves likely evolved metamorphosis independently [12,17-24]. Still, a more complete understanding of the mechanisms underlying echinoderm metamorphosis can shed new light on the origins of metamorphosis in general [20]. Furthermore, a better mechanistic appreciation of echinoderm metamorphosis will provide insights into body plan evolution within the echinoderms and ultimately the deuterostomes [25-28]. Finally, the metamorphic transition is notable as one in which multiple external environmental signals are integrated by the developing larvae to control the proper timing and location of settlement [12], which subsequently impacts the successful recruitment of larvae into benthic populations. Therefore, there is a fascinating interplay between ecology, evolution and development that occurs at the metamorphic transition in marine invertebrates that makes it an under-appreciated archetype for eco-evo-devo studies (sensu [29,30]).

One impediment to using echinoderms as subjects for detailed metamorphic studies is the lack of an agreed upon, simple, yet appropriately detailed scheme for describing the stages of juvenile morphogenesis leading up to settlement (except for brittle stars; [31,32]). This information gap exists despite many careful studies over more than a hundred years into the ontogeny of the structures in echinoderm larvae that are fated to form the juvenile (i.e., juvenile structures in contrast to larval ones – see e.g., [33-36]).

Two explicit staging schemes have been proposed for echinoids that include the development of the juvenile rudiment through settlement: one for purple urchins [37], the other encompassing three other regular echinoids [38]. Unfortunately, these schemes provide little detail on the events of juvenile morphogenesis that occur after the onset of juvenile calcification, a period of complex ontogeny which represents approximately the last 1/3 of the total larval phase, and during which most of the definitive structures of the juvenile first appear. Indeed, these are the very stages where we expect to find species-specific differences in ontogeny underlying the substantial variation in juvenile morphology across echinoids [39]. Furthermore, the proposed purple urchin staging scheme of Smith et al. [37] mixes larval and juvenile characters. This is a considerable shortcoming, as sea urchin larvae demonstrate marked phenotypic plasticity with respect to the timing of appearance of larval versus juvenile structures [38,40,41].

In an effort to build on these existing schemes for sea urchin larval development, we here propose the first detailed staging scheme in any echinoid for the morphogenesis of juvenile structures alone, from rudiment invagination until settlement. We have divided our scheme into soft tissue and skeletogenic stages, in part for clarity, and in part due to our observations of variation within and among clutches in the timing of appearance of skeleton relative to our defined soft tissue stages. In fact, such heterochronies (differences among individuals in the relative timing of developmental events), occur commonly in the formation of different juvenile characters, and we have endeavored to take account of this variation in our proposed staging scheme. Above all, we have only included characters that are readily visible in live larvae using even the most basic compound microscope equipped with cross-polarized light at about 100× total magnification. Our intention is that this staging scheme will not only facilitate a broader range of studies into late larval development in purple sea urchins, but will also be the starting point for similar staging schemes across echinoids and other echinoderms.

## Results

### Staging scheme

**Figure 1:**
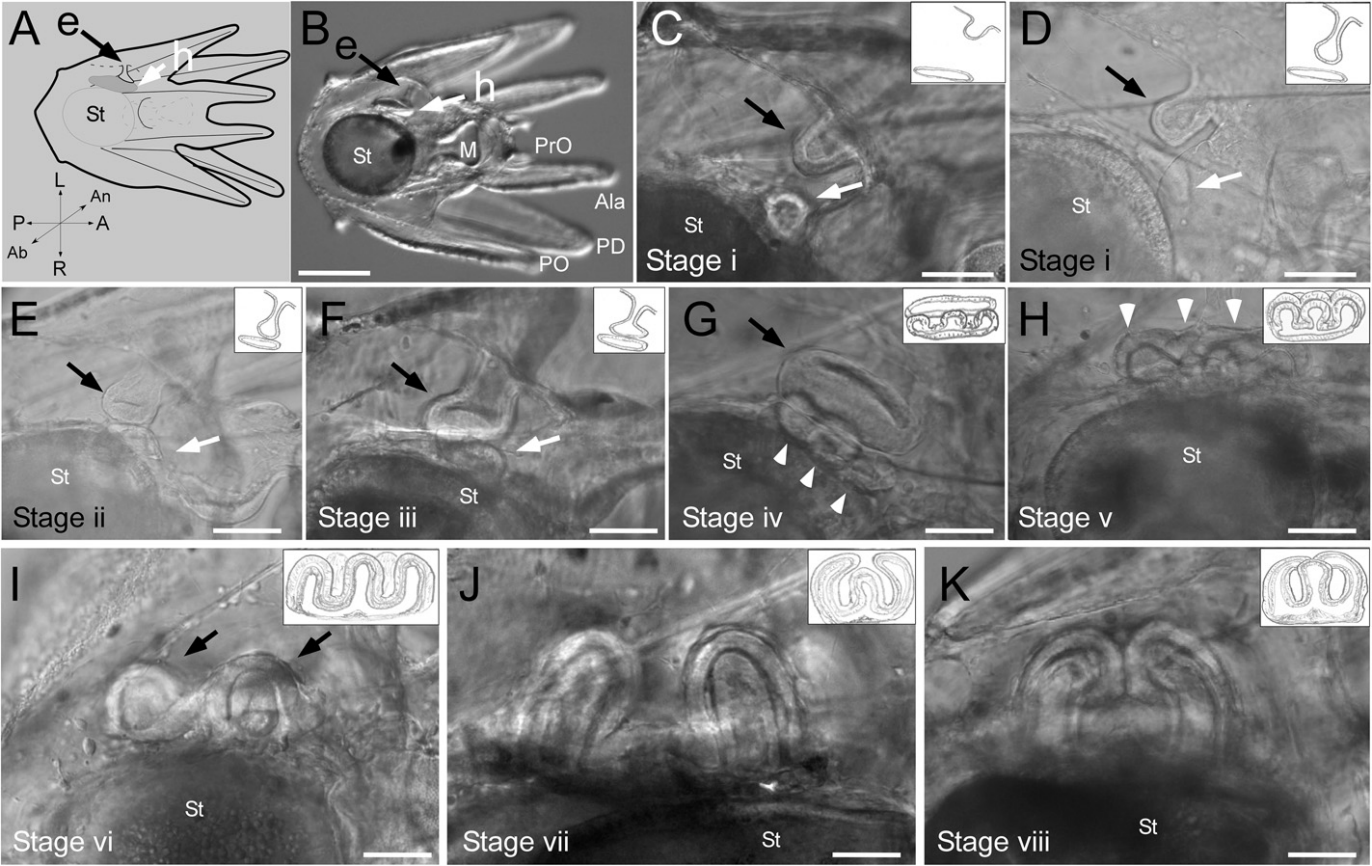
**Examples of soft tissue developmental stages of *S. purpuratus *larvae as defined in Table**1**(roman numerals).** All images (except as indicated) are close-up views of the rudiment in the same orientation as indicated in **A**, i.e., an abanal view of the larvae (sensu [37]). Insets in **C-K** show schematic views of the stages in question; see also Table 1. **(A)** Overview diagram of larva with corresponding DIC image of actual larva (**B** – anal view). **(C-F)** Black arrow- invaginating ectoderm (e); white arrow- hydrocoel (h). **(C)** Stage i, approximately 60% ectodermal invagination. **(D)** Stage i, approximately 90% ectodermal invagination (anal view). **(E)** Stage ii, contact of invaginating ectoderm with hydrocoel (anal view). **(F)** Stage iii, ectoderm flattening alongside hydrocoel (anal view). **(G)** Stage iv, 5-fold mesoderm (first visible sign of 5-fold symmetry); white arrowheads- 3 of the 5 primary podia anlage are visible in this view; black arrow- invaginating ectoderm. **(H)** Stage v, 5-fold ectoderm; arrowheads as in **E**. **(I)** Stage vi, primary podia stage, arrowheads indicate two of the forming podia. **(J)** Soft tissue Stage vii with folded primary podia. **(K)** Soft tissue Stage viii with primary podia bending at the tip and touching each other (anal view). Note that schematic in **A** does not show the pair of preoral arms for simplicity. Abbreviations: Ala – anterolateral arms; M – Mouth; St – stomach; PD – postdorsal arms; PO – postoral arms, PrO – preoral arms; h – hydrocoel; e – invaginating ectoderm (=vestibule); L – Left larval side; R – Right larval side; A – anterior; P – posterior; An – anal; Ab – abanal. Scale bars: **B**- 200 μm **C**, **D**, **E** – 35 μm; **F** - 25 μm; **G**, **H**, **I** - 40 μm, **J** - 30 μm; **K** - 40 μm.

**Figure 2:**
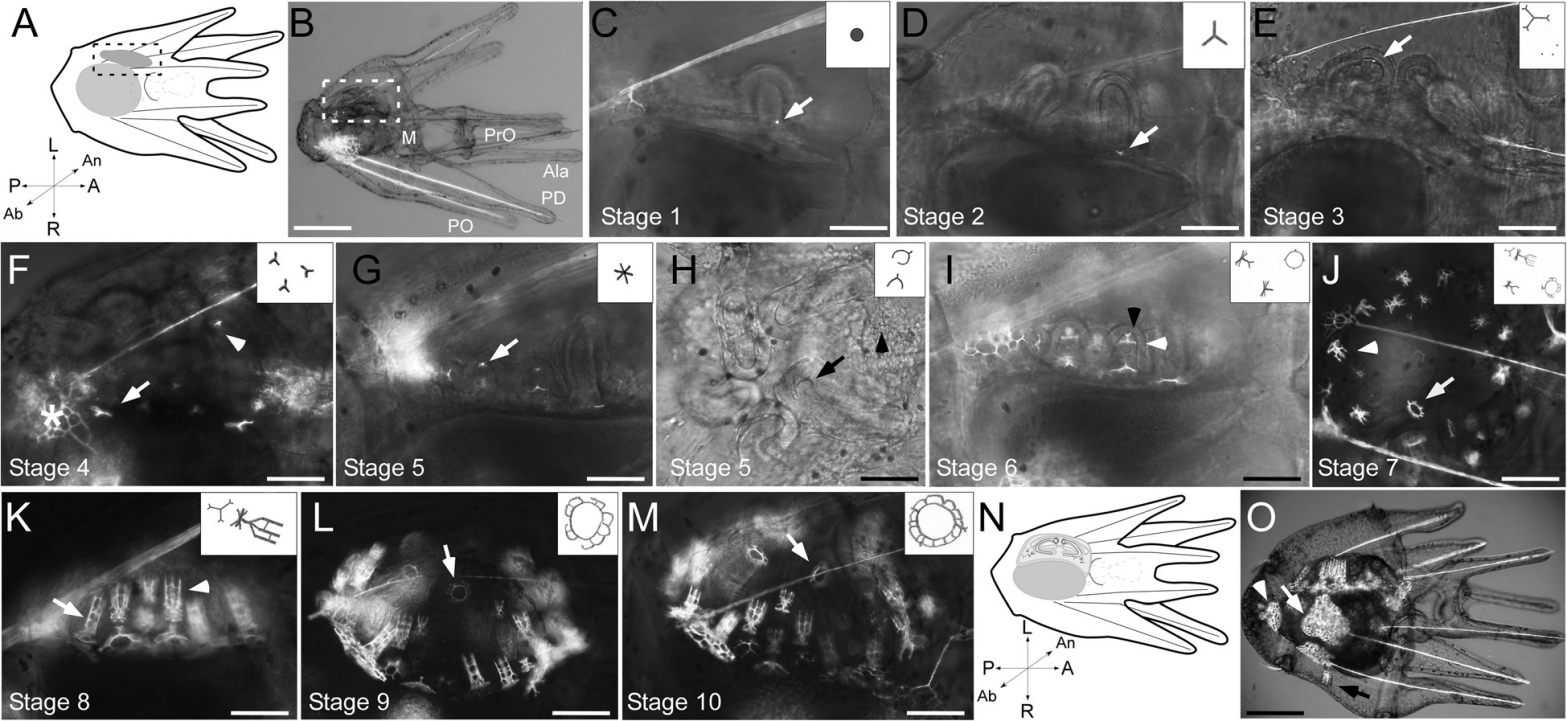
**Examples of skeletogenic developmental stages of *S. purpuratus *larvae as defined in Table**2**(arabic numerals).** All images (except as indicated) are rudiment close-up views in same orientation (i.e., abanal view, sensu [37]) as indicated in **A** (earlier stages) and **N** (later stages). **E**, **F**, **G**, **K**, **M** are anal views. Insets in **C-M**: schematic views of defining skeletogenic features for stage in question, not drawn to scale; see also Table 2. Drawing **(A)** and corresponding live image **(B)** of representative early skeletogenic stage larva. **(C)** Stage 1: spicule dot (white arrow). **(D)** Stage 2: spicule (white arrow). **(E)** Stage 3: tube foot spicule dot (white arrow). **(F)** Stage 4: tri-radiate tube foot spicule (white arrowhead); multi-branched spicule (white arrow), fated to form ocular plate 1 (see [34,35]). White asterisk indicates ocular plate 5 –a non-rudiment skeletal character not included in our scheme– which forms off of the left PO rod (see [43]). **(G)** Stage 5: spine primordium (6-sided star- white arrow; note also spine lumen visible at this and later stages). **(H)** Also Stage 5: incomplete TF ring (black arrow); multi-branched spicule indicated (black arrowhead) is fated to form the interambulacral skeleton at the base of an adult spine in interambulacrum 1 (sensu [34,35]; but see [44]). Note this view is looking down on the left side of larva. **(I)** Stage 6: spine primordium (white arrowhead) with base of spine (black arrowhead) extending orthogonal to that. **(J)** Stage 7: pre-spine (fronds present with no cross hatches; white arrowhead), and TF ring with 2nd ring <1/2 complete (white arrow). Larva viewed from left side. **(K)** Stage 8: adult spine with cross hatches (white arrowhead); juvenile spine (white arrow) indicated for comparison; anal view. **(L)** Stage 9: TF ring with 2nd ring >1/2 complete (white arrow is pointing directly to a gap in 2nd ring). **(M)** Stage 10: TF ring with 2nd ring complete (white arrow); anal view. **(N)** schematic of larva as seen in panels I-M with larger rudiment and spines becoming recognizable. **(O)** Stage 10. Whole larva (oriented as in **N**) showing, in addition to the rudiment, several of the non-rudiment juvenile skeletal characters that we do not incorporate in our staging scheme (see also Additional file 1: Figure S1 and [43]): genital plate 5 (white arrow), posterior juvenile spines articulating with genital plate 4 (white arrowhead) and right side posterior juvenile spine (black arrow; see [45]). Ocular 5 is indicated in panel **F**. Note that schematic does not show PO arms for simplicity. Abbreviations: Ala – anterolateral arms; M – Mouth; St – stomach; PD – postdorsal arms; PO – postoral arms, PrO – preoral arms. Scale bars **B** – 300 μm; **C-D**: 70 μm; **E**- 80 μm; **F-M** – 70 μm; **O** - 200 μm

In Table 1 & Figure 1 (for soft-tissue stages) and in Table 2 & Figure 2 (for skeletogenic stages) we present a new staging scheme for the development of juvenile structures in the purple sea urchin, *Strongylocentrotus purpuratus*, beginning with the invagination of the larval epithelium that forms the juvenile ectoderm (=“echinus rudiment”), and ending with well-developed tube foot end plates and the elongation of adult spines (reviewed in [42]). This latter stage (our Stage 10 in Table 2, Figure 2) is approximately the point at which larvae become competent to settle when reared in the laboratory under controlled conditions (see Figure 2M & O), and occurs at approximately 4 to 4.5 weeks after fertilization at 14°C under abundant food conditions.

**Table 1:** Soft tissue stages in rudiment

**Stage**	**Shorthand**	**Days**	**Drawings**	**Smith et al. 2008 [**[37]**]**	**Chino et al. 1994 [**[38]**]**	**Description**
-	pre invagination	< 13		I-II	a-c	6-8 arm larva, no invagination forming on the left side yet.
i	invagination	14		III	c-d	Rudiment invaginating on left side, not yet contacting hydrocoel; this can be further subdivided as “% invagination” (i.e., the depth of the invagination measured from the ectoderm through the axis of the invagination to the hydrocoel).
ii	contact	15		IV	e-f	Rudiment has contacted the hydrocoel, but has not flattened alongside it yet.
iii	contact flattened	16		IV	f-g	Invaginated rudiment has now flattened alongside the hydrocoel, but there is as yet no 5-fold symmetry apparent in the mesoderm or the invaginated ectoderm.
iv	5-fold mesoderm	17		V	h	Hydrocoel showing first visible signs of 5-fold symmetry (5 bumps), but the ectoderm (invaginated rudiment) not yet showing any sign of 5-fold symmetry.
v	5-fold ectoderm	18-19		V	i	Ectoderm now also showing 5-fold symmetry as the primordia of the 5 podia begin to push through the floor of the vestibular ectoderm. At this point, the interior of the 5 incipient podia are spherical in shape or shorter than wide.
vi	primary podia (pp)	19-21		V	i-j	Interior of 5 primary podia are now taller than wide, but the podia are not yet folding in towards one another.
vii	pp-folded	21-23		VI?	k	The 5 podia are now folding towards one another (i.e. towards the center of the oral field of the forming juvenile), but the tips of adjacent podia are not yet touching one another. This soft tissue stage usually coincides with skeletogenic Stage 1 or 2 (see Table 2).
viii	pp-touching	23		VI	k-l	The tips of at least 2 of the 5 tube feet are now touching. This soft tissue stage usually coincides with skeletogenic Stage 2+ (see Table 2).

Description and illustration of stages presented in the new staging scheme as well as approximate timing in days after fertilization at 14°C. Note that roman numerals in the column “Smith et al. [37]” and the letters in the column “Chino et al. [38]” are the stages proposed in those studies (see Figure eleven in [37], and Figure two in [38]) as they relate approximately to the stages used in our new soft tissue staging scheme here. Question marks indicate ambiguities in the comparisons between staging schemes.

**Table 2:** Skeletogenic stages in rudiment

**Stage**	**Shorthand**	**Days**	**Drawings**	**Smith et al. 2008 [**[37]**]**	**Chino et al. 1994 [**[38]**]**	**Description**
0	no skeleton	< 21		I-V	a-j	No spicule dot (or any other rudiment skeleton) is visible; “soft tissue” Stage vi or earlier (see Table 1).
1	spicule dot	22		VI	k	At least one spicule dot is visible in the rudiment (first spicule dot is usually at the anterior edge of the rudiment, near the stomach/esophagus border); this occurs at about “soft tissue” Stage vi-vii (see Table 1).
2	spicule	22-23		VI	k	At least one triradiate spicule present in the rudiment.
3	MB/TF spic dots	23		VI	k	Multi-branched spicule (MB) and/or tube foot (TF) spicule dots present in the rudiment. The first MB spicules seen are the de novo ocular plate primordia of the urchin test, as well as the interambulacral plate primordia that will articulate with adult spines after Stage 8.
4	TF spicules	24		VI	k	TF triradiate spicule present in at least one primary podium.
5	sp primord/incomplete ring	25		VI	k-l	Adult spine primordium (6-prong spicule) present and/or TF spicules elongating but not forming complete ring in any primary podia.
6	spine primord + base/1st TF ring complete	26		VI	l	Adult spine primordium element elongating in direction orthogonal to 6-sided primordium (i.e., along the axis of the incipient spine) and/or 1st TF ring has fused to form a complete ring in at least one primary podium. 2nd TF ring may be beginning to form as bifurcating extensions from the 1st TF ring, but none of these bifurcations have yet started to fuse back with the 1st TF ring.
7	pre-spine/2nd TF ring < ½ complete	27		VI	l	At least one adult spine primordium has a complete base + 6 fronds, but no cross hatches have formed between the fronds and/or the 2nd TF ring has bifurcating spicules that may have started to fuse in at least one primary podium, forming part of the incipient second ring, but this ring is at most half complete.
8	spines	28		VI	l	At least one cross hatch is now present in at least one forming adult (6-sided) spine. Spine growth can be further subdivided by counting the average and maximum number of cross hatches (see Figure 5).
9	2nd TF ring > ½ complete	29		VI?	l	Bifurcating bits of the 2nd TF ring have continued to fuse in at least one primary podium, so that more than half or the 2nd TF ring is complete, but the 2nd TF ring is not yet complete in any podium.
10	2nd TF ring complete	30		VII?	m	2nd TF ring is now complete in at least one primary podium. Larvae start becoming competent to settle at around this stage, though timing of competence seems to vary with skeletogenic stage.

Description and illustration of stages presented in the new staging scheme as well as approximate timing in days after fertilization at 14°C. Note that roman numerals in the column “Smith et al. [37]” and the letters in the column “Chino et al. [38]” are the stage designations proposed in those studies (see Figure eleven in Smith et al. [37], and Figure two in Chino et al. [38]) as they relate approximately to the stages used in our new skeletogenic staging scheme here. Question marks indicate ambiguities in the comparison of staging schemes.

We have separated the proposed scheme into soft tissue stages (roman numerals in Table 1, Figure 1) and skeletogenic stages (arabic numerals in Table 2, Figure 2), as we have noticed heterochronic variation among and within clutches in the soft tissue stage at which skeleton is first visible. We show representative images of the defining features of the various stages in Figures 1 & 2, and provide sketches of the same in Tables 1 & 2 that are also shown in Figures 1 & 2 as insets.

All of the features that we used to define the various stages are readily visible in whole mounts of live larvae with raised cover glass, and employing any compound microscope at approximately 100× total magnification. For this reason, we did not include any characters (such as the developing epineural folds and dental sacs) that typically require histological analysis for their visualization – development of a companion histological staging analysis would be a useful expansion on the staging scheme presented here. By contrast, the use of cross-polarized light (placing a polarizing filter on either side of the sample and rotating one filter relative to the other to quench the light) results in birefringence of larval and juvenile skeleton, allowing for visualization of skeletal elements deep within the rudiment of live larvae.

Several of our skeletogenic stages (Table 2, Figure 2) include more than one defining character. For example, Stage 3 is defined as having multibranched spicules and/or tube foot spicule dots present. The reason that we have combined characters in this way is because we have noted heterochronies in some larvae in the relative appearance of the two classes of characters. Therefore, in this example, we score a larva as Stage 3 if either one or more multibranched spicules or one or more tube foot spicule dots (or both) are visible in that larva. If required for specific analyses, these characters can be easily analyzed in isolation.

Although we examined juvenile skeletal structures that form both inside and outside of the juvenile rudiment, our staging scheme itself includes only juvenile skeletal structures (the interambulacral plates, spines, tube feet and the subset of ocular plates) that form inside of the rudiment itself. The reason that we chose to exclude any extra-rudiment skeleton from our staging scheme is that their ontogenic progression was heterochronic with respect to the rudiment skeleton in different larvae, even within the same culture vessel (see Additional file 1: Figure S1). As such, the inclusion of extra-embryonic skeletal structures in our staging scheme would have been non-informative alongside the easily visible and characterizable rudiment skeleton that we did include. Furthermore, as we demonstrate below, each of our stages as defined is non-ambiguous and well-spaced (approximately 24 hours of development at 14°C between stages), and thus the characters we include seem sufficient in order for any researcher to quickly and repeatedly characterize the stage of live individual larvae examined in a standard compound microscope.

### Overview of the proposed scheme

A brief summary of all developmental stages is provided here, with details on each soft tissue and skeletogenic stage (as well as approximate timing at 14°C with abundant food) provided in Tables 1 & 2, respectively. The juvenile rudiment begins to form with an ectodermal invagination (the vestibule) on the left side of the larva (Stage i; Figure 1C, D), which contacts (Stage ii; Figure 1E) and then flattens against the left hydrocoel (Stage iii; Figure 1F). Soon thereafter, the rudiment shows the first signs of the adult pentameral symmetry as five bumps in the hydrocoel, pressed against the floor of the vestibule (Stage iv; Figure 1G). As these five bumps continue to penetrate the overlying vestibule, the vestibular ectoderm itself begins to reveal a pentameral pattern (Stage v; Figure 1H). During subsequent days, the five bumps (which we now refer to as the “primary podia”) lengthen (Stage vi; Figure 1I), begin to curve in towards one another (i.e., towards the oral field of the rudiment; Stage vii; Figure 1J), and ultimately the tips of the primary podia touch one another (Stage viii; Figure 1K).The first skeletal elements of the juvenile are visible in soft tissue Stage vi or vii as small spicule dots usually visible near the esophagus/stomach border (Stage 1; Figure 2C). These will develop into triradiate spicules (Stage 2; Figure 2D), and then into multi-branched spicules (Stage 3; Figure 2E), the first of which are ocular plate and interambulacral plate primordia. At this same stage, skeletal spicule dots appear at the tips of each of the primary podia, which then become tri-radiate spicules (Stage 4; Figure 2F). These spicules then begin to elongate from two of the spicule arms (Stage 5; Figure 2G) to begin to form the first ring of the tube foot end plates. At this same stage, 6-sided spicules appear (Stage 5; Figure 2H), which are the primordia of the adult spines. These spicules elongate along the axis orthogonal to the original 6-sided spicule, forming the base of the incipient adult spine, at around the same time that the first tube foot skeletal rings are complete (Stage 6; Figure 2I). In the next stage (Stage 7; Figure 2J), the spine primordia continue to elongate into 6 parallel fronds lacking any cross hatches, while the second concentric tube foot skeletal ring begins to form. Stage 8 (Figure 2K) is characterized by the formation of the first cross hatch (perpendicular to and connecting two adjacent spine fronds) in at least one of the adult spines. The final two stages are defined by tube foot skeleton where the second concentric ring is >1/2 complete (Stage 9; Figure 2L) and then complete (Stage 10; Figure 2M).

### Developmental timing

**Figure 3:**
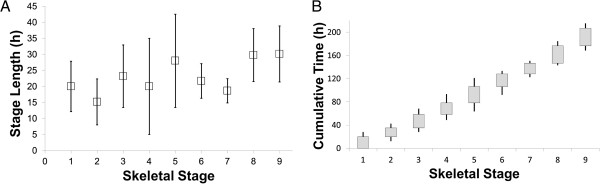
**Temporal progression of skeletal development in *S. purpuratus *larvae. A)** Mean stage length as a function of skeletal stage. Error bars are one standard error of the mean. **B)** Cumulative time as a function of skeletal stage, starting at the onset of Stage 1 (first appearance of any skeleton in rudiment; see Table 2). Boxes indicate the approximate cumulative time interval during which a larva is in a given stage; error bars indicate 95% confidence interval.

By culturing individual larvae in well plates, and staging them at 0, 24 and in some cases 48 hours, we were able to make estimations of the length in hours for each of our skeletogenic stages (see Figure 3A). From these data, we plotted cumulative time through the ten skeletogenic stages in Figure 3B.We caution readers that the data that we present in Figure 3 are intended mainly for heuristic purposes; we note substantial variation among larvae (and among experiments) in the length of given stages. With that caveat in mind, Figure 3A indicates that most of our proposed skeletogenic stages are approximately 24 hours in length at 14°C.

In order to test whether our temporary mounting methodology for viewing live larvae injured them, we compared developmental rates in mounted and unmounted larvae in a separate experiment in Guelph. On average, mounted larvae progressed 2 stages (from Stage 4 to Stage 6; see also Figure 2) during the 48 hour period of the experiment. The skeletogenic stage for mounted larvae at the end of the 48 h period was 6 ± 1.3 (Std) compared to 6 ± 1.3 (Std) for un-mounted larvae. We detected no statistical difference between the two treatments using a 2-tailed independent sample *t*-test (t_1,19_ = -0.53; p = 0.60). Converting these stage values into time (using the cumulative time data presented in Figure 3B) did not change this result (data not shown).

### Robustness of the staging scheme

**Figure 4:**
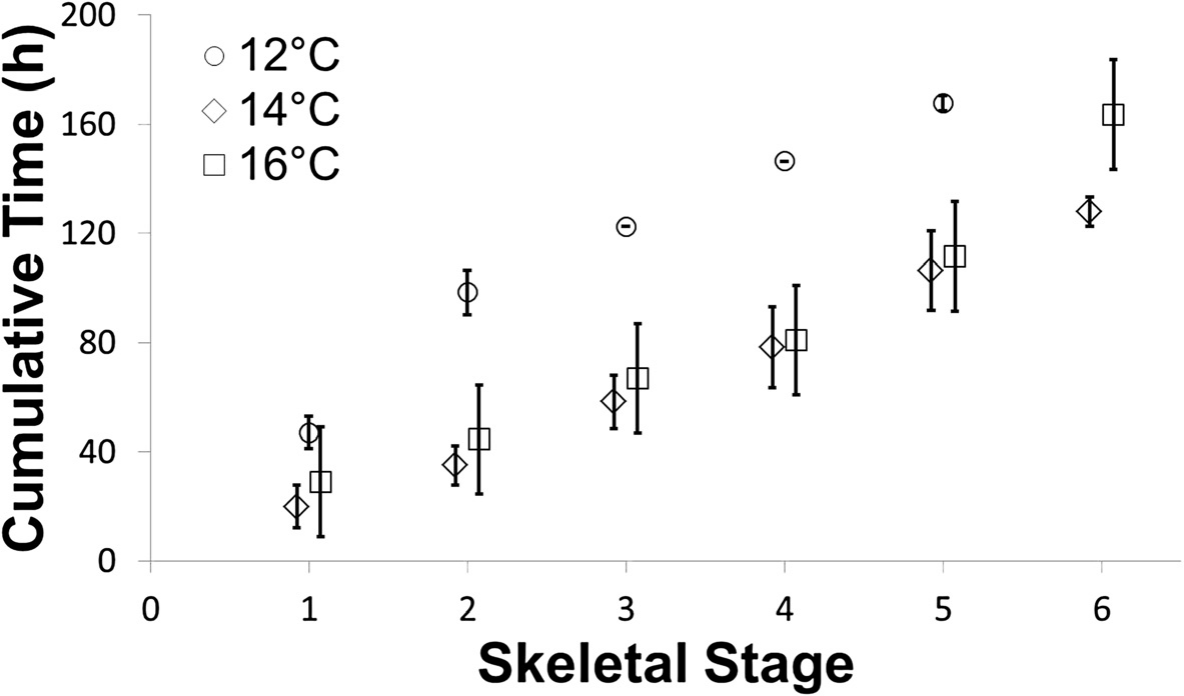
**Comparison of cumulative time to reach specific stages at three different temperatures.** The temporal progression through these stages was clearly slowest at 12°C, whereas no difference is apparent between 14°C and 16°C. Note that the data for 12°C and 16°C originate from the Seattle dataset while the data for 14°C originate from the Guelph dataset. Several other differences existed between these two experiments (see Methods). Error bars indicate 95% confidence interval.

To test whether our proposed staging scheme is robust under a variety of environmental conditions, we repeated our well plate experiment with larvae that had been reared in natural (as opposed to artificial) sea water, with lower food levels (but still likely *ad libitum* - [46,47]) and at a higher temperature (16°C) – and that derived from a different source population, widely separated from the California population of urchins studied in Guelph. In this ‘Seattle’ experiment, we repeated our well-plate individual larval rearing methodology at two temperatures: 12°C and 16°C.Figure 4 shows a summary of the Seattle experiment, plotted alongside our main (‘Guelph’ experiment, 14°C) dataset. First, the range of rearing conditions and temperatures examined did not alter the relative progression of stages. Second, lower temperature rearing (12°C) resulted in slower overall progression through the skeletogenic stages. And third, our 16°C Seattle larvae progressed through their stages at a similar rate as did the Guelph 14°C larvae. Among other possible interpretations, this latter result might indicate that the higher food levels that we used in Guelph compensated for the lower temperature, or that 15°C is the optimum rearing temperature, and that 14°C and 16°C lie on either side of that optimal development peak.

In addition to this Seattle well plate experiment, we have reared multiple batches of sea urchin larvae through settlement in 5 different locations with different adult feeding and sea water conditions (see Methods). During each of these rearings, we have tracked the stages of our developing larvae and have not noticed any variations from the staging scheme that we propose here.

### Spine growth

**Figure 5:**
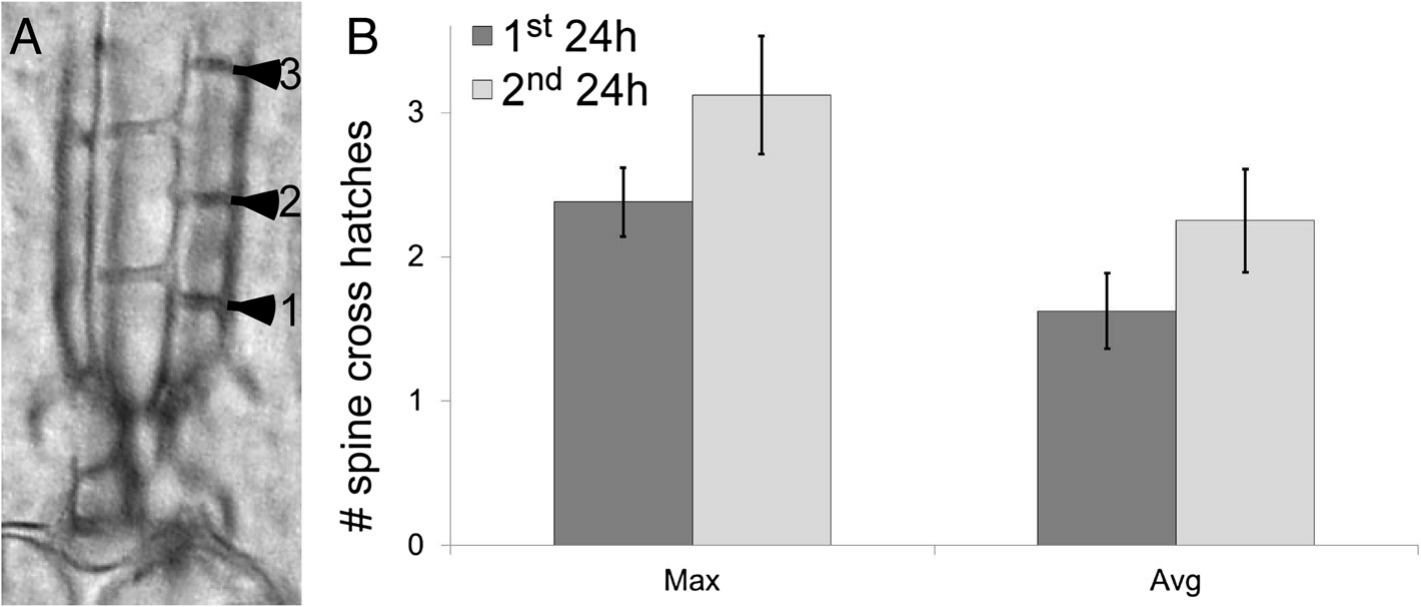
**Spine elongation in *S. purpuratus *rudiment. A)** single developing adult spine with cross hatches; this spine has 3 cross hatches on the indicated face, but notice that other faces have fewer. **B)** Spine elongation measured by the average (Avg) and maximum (Max) number of cross hatches in the adult spines of developing *S. purpuratus* larvae at Stage 8 or later (see Table 2). We detected no significant difference in spine elongation between the first and second 24 hours of development in well plates during the course of the experiment at 14°C. Error bars are one standard error of the mean.

We are aware that the skeletogenic features that we have used in our staging scheme are more qualitative than quantitative in nature. Nevertheless, there is one quantitative character that is easily scored in late stage larvae: the elongation of the growing spines. As the incipient spines increase in length, they increase the number of cross bars that form between the adjacent spine fronds in a step-wise, linear fashion (Figure 5A). Therefore, one can use the number of cross bars (=cross hatches) present in a developing spine as a numerical proxy for spine length.

Figure 5B shows the increase in the mean (Avg) and maximum (Max) number of cross hatches in our larvae during the first and second 24 hours of development in our well plate set-up at 14°C (Guelph experiment). Two conclusions are apparent from these data: 1) spines grew in our well plate experiment at a rate of about 2 cross hatches per day; and 2) there is no significant difference in the rate of spine growth when comparing the first and second 24 hours of our experiment (Max: t_38_ = -1.7; p = 0.10; Avg: t_38_ = -1.4; p = 0.16). If anything, the results in Figure 5B indicate a trend towards faster spine growth (Avg and Max) in the second 24 hours.

## Discussion

Metamorphosis is a widespread phenomenon across animals and non-animals alike [12,18,48,49]; in the most extreme examples, the pre-metamorphic form (e.g., larva) is so disparate from the post-metamorphic form (e.g., juvenile, adult) that biologists once classified them as being entirely unrelated (e.g., [50]). As such, metamorphosis is a period where complex ontogenetic processes occur in a relatively short time [12,14,18,48,49], a phenomenon not unlike what occurs during embryogenesis.

Many echinoderms (sea urchins, sea stars, sea cucumbers and their kin) have life histories containing notable examples of such a radical metamorphosis, where their bilaterally-symmetric larva transforms in relatively short order to a pentameral adult, a process that has fascinated biologists for over a hundred years. Despite this interest, we still lack a thorough mechanistic understanding of metamorphosis in any echinoderm, or indeed in any marine invertebrate. Thus while the purple sea urchin, *Strongylocentrotus purpuratus*, has progressed in recent years from being a useful subject for classical embryogenesis studies to revealing detailed molecular genetic mechanisms of development, a parallel advance in our understanding of its ‘second embryogenesis’ [51] –metamorphosis– has not materialized.

The lack of a clearly defined, sufficiently detailed, and broadly accepted staging scheme for commonly studied echinoderms (but see [31,32]) is one explanation for the lack of parallel progress in understanding their metamorphoses. We thus offer the purple urchin staging scheme described herein as a step to overcoming this deficit, and thus facilitating a broader range of investigations into sea urchin (and ultimately echinoderm) metamorphosis. In this sense, our staging scheme was inspired by similar ones that remain widely used in *Drosophila*[52], the sea hare *Aplysia californica*[53], *Xenopus*[54] and zebrafish [55], and like ours, are focused on characters readily visible under the microscope in live individual embryos and larvae.

### Comparison with previous echinoid staging schemes

Homologs of the characters that we focused on for the majority of our stages had been previously described in detail by Macbride [56-59], von Ubisch [60] and Gordon [34,35] among others in their classic histological studies of pre-settlement juvenile development in a variety of echinoid taxa. Nevertheless, a treatment of these skeletal characters is largely absent from the purple urchin staging scheme of Smith and colleagues [37], and from the echinoid staging scheme of Chino and colleagues [38] as well. As such, both of these latter schemes compress the final 1/3 of larval development (or approximately 10 days in *S. purpuratus*) into 2–3 stages (as compared to our 10 stages; see Table 2). This seems insufficient given that this period represents the bulk of juvenile morphogenesis in the larva, and is thus when the majority of adult structures first make their appearance during ontogeny. By contrast, many of our proposed rudiment soft tissue stages are similar to those proposed by Smith and colleagues [37] and Chino and colleagues [38], as we indicate in Table 1. For this reason, and because of the detailed histological descriptions of juvenile purple urchin morphogenesis in Smith et al. [37], we see our proposed staging scheme as broadly complementary to these previous efforts.

### Phenotypic plasticity and heterochronies

The biggest concern in proposing any staging scheme for echinoderm larvae is taking account of the substantial phenotypic plasticity in their ontogeny [61]. For this reason, we specifically avoided combining, in our proposed staging scheme, larval and juvenile characters, the developmental trajectories of which can vary substantially relative to one another depending on food and other rearing conditions [38,40,41,62,63].

Nevertheless, even when restricting our analysis to juvenile characters alone, we noted instances where different larvae exhibited heterochronies: namely, variation in the relative appearance of distinct characters. One example is the juvenile spines that develop on the right side of purple urchin larvae, outside of the rudiment proper (see [37,45]); another is the genital plates of the test, several of which form as bifurcations and proliferations off of larval skeletal rods [see 34,35]. We left an analysis of those structures out of our staging scheme since we observed substantial heterochronic variation in their developmental trajectories relative to rudiment characters, and thus their inclusion would not be informative (see Additional file 1: Figure S1). We also noted much more subtle heterochronies in some of the characters that we did include; in such cases (see skeletogenic Stages 3 and 5–7), we accounted for the variation by combining more than one character into a single stage. Likewise, because we noticed heterochronic variation (even within clutches) in the soft tissue stage at which we first observed juvenile skeleton formation, we split our staging scheme into two sub-schemes: one for soft tissue characters, the other for skeletogenic characters.

To address the issue that variation in rearing conditions might likewise lead to heterochronic shifts in the ontogeny of different juvenile characters, we reared larvae under five conditions that differed in sea water chemistry (artificial versus natural), food level, temperature, method of culture mixing and source population. Despite rearing larvae with these multiple sources of variance, we did not observe any heterochronies in our staging characters. Therefore, we are confident that the broad outlines of our staging scheme should hold across *S. purpuratus* populations and in different rearing conditions.

### Applicability outside of *S. purpuratus*

One of the great advantages in studying echinoid larval development and metamorphosis is the great diversity in developmental modes, larval forms and adult habitats within a group having an excellent fossil record and robust phylogenetic hypotheses for their relationships [26,64,65]. This suite of features makes echinoids prime candidates for a wide range of comparative studies on larval development and ecology, metamorphosis and settlement, as well as juvenile form, function and behavior.

To encourage the use of echinoids in these ways, we envision our staging scheme as a template for the development of similar schemes across echinoids. Over 120 years of research effort has been directed at descriptions of late larval and juvenile development across a wide range of echinoid species [12,34-40,42,56-60,66-89] and from these studies it is clear that there is substantial variation across echinoids in what and when juvenile skeletal elements form during late larval development. For example, pedicellariae form before settlement in a widely divergent assemblage of species (reviewed in [89]), whereas they form only after settlement in most taxa, including *S. purpuratus*. Emlet [39] undertook a comparison of the juvenile structures present shortly after settlement in a disparate group of 31 echinoid species, revealing substantial variation in early juvenile form, variation that must have been generated during late larval development. The staging scheme proposed here can be explicitly used to trace the ontogenetic basis for this variation.

Specifically, we predict that such comparative studies will uncover both heterochronies (see [90]) and heterotopies (change in the relative position of developmental events) in the formation of juvenile structures in late larvae, changes that might be hypothesized to be related to selection – or otherwise reveal constraints – on juvenile form and function. Indeed, many of the descriptive studies cited have already indicated the existence of this kind of variation; with an explicit staging scheme to use for comparisons, such as the one developed here, such variation can be easily demonstrated.

We would also advocate that our general approach used herein be applied to non-echinoid echinoderms as well, enabling more precise comparisons among and between the different classes of echinoderms. Such a broadening would help harness the power of the comparative approach for studies ranging from life history evolution to functional morphology of larvae and juveniles to the evolution of body plans in the remarkably diverse echinoderm phylum.

## Conclusions

Here we present a novel, detailed staging scheme for late larval development for the purple sea urchin, *Strongylocentrotus purpuratus*. We conceived this scheme to allow sea urchin researchers to quickly and consistently identify the stage of live larvae during the period of rapid juvenile morphogenesis that occurs in the final weeks before settlement, using standard microscopy techniques. We offer a detailed comparison of our scheme to previous efforts, providing a side by side comparison of corresponding stages. This analysis reveals that our new staging scheme provides a more detailed picture of juvenile development using ontogenetically informative characters. Inspired by widely used schemes in other organisms such as *Drosophila*, zebrafish and *Xenopus*, our proposed staging scheme provides both a useful method to study late larval development in the purple urchin, and a framework for developing similar staging schemes across echinoderms. Such efforts will have a high impact on evolutionary developmental as well as larval ecology studies and facilitate research on this important deuterostome group.

## Methods

We conducted staging observations on multiple clutches of purple sea urchin (*Strongylocentrotus purpuratus*) larvae from 2010–2014, fertilized and reared through settlement in the laboratory using our modifications of standard methods; [3] details below). In order to insure consistency of our staging scheme, we reared larvae in 5 different locations in North America (SEA-Seattle, WA; FHL-Friday Harbor Labs, WA; BML-Bodega Marine Laboratories, CA; HMS-Hopkins Marine Station, CA and UOG-Guelph, ON).

### Urchins and spawning

Adult urchins derived from two distinct USA source populations: Slip Point (Clallam Bay, WA; SEA, FHL) and The Cultured Abalone Ltd (Goleta, CA; UOG, BML, HMS). At UOG, we maintained urchins in the Hagen Aqualab, University of Guelph, in artificial seawater (Instant Ocean™; Instant Ocean) at 12°C and 34 ppt salinity. We fed the urchins rehydrated kombu kelp (*Laminaria sp.*) *ad libitum*. For the SEA and FHL rearings, we used urchins maintained in subtidal cages suspended off the floating docks at FHL, fed throughout the year *ad libitum* with drift kelp (mainly blades of *Nereocystis leutkeana*).

For the HMS and BML rearings, we used urchins maintained in the dark in flow-through natural sea water tables at HMS, fed throughout the year *ad libitum* with giant kelp (*Macrocystis pyrifera*).

We spawned adult sea urchins by gentle shaking or intra-coelomic injection with 0.5 M KCl, and fertilized spawned eggs (>90% fertilization success) with diluted sperm using standard methods [3].

### Larval culturing

For all embryo and larval culturing in Guelph, we used 0.2 μm Millipore™-filtered artificial seawater (MFASW), sterilized with UV light. We cultured embryos at 14°C until hatching (24 hours) at which point we poured off swimming embryos and set up our main cultures at 1 larva/4 ml derived from one male and one female. We used a mechanical stirring system [3] to keep larvae in suspension, and fed them a mixture of *Dunieliella tertiolecta* (12 cells/μl) and *Rhodomonas lens* (6 cells/μl). We changed greater than 95% of the water every two days by reverse filtration, and fed the larvae as above.

For our embryo and larval rearings at FHL, SEA, HMS and BML, we derived our cultures from equal-part mixtures of single-parent fertilizations [either 3 males × 1 female (3M × 1F), 3F × 1M or 2F × 2M] at an initial density of 1 embryo/ml, and maintained either using a mechanical stirring system (FHL, HMS, BML; 3]) or a shaking water bath (SEA) to keep larvae in suspension. Starting on day 3, we fed larvae a mixture of *Dunieliella teriolecta* (3 cells/μl) and *Rhodomonas spp.* (2.5 cells/μl) every 2 days following water changes as described above. We reduced the larval density to 0.1-0.25 larvae/ml on about day 16 (6 arm plutei, before rudiment invagination). At FHL and BML, we cultured our larvae at sea table temperatures, which vary from an average of 10-14°C depending on the time of year. At HMS and SEA, we maintained our larvae at constant temperatures (14°C and 16°C, respectively).

### Staging scheme

To develop the staging scheme, we identified dozens of score-able soft tissue and skeletogenic juvenile characters (see also [88]), and characterized hundreds of total larva for the presence or absence of that character. Our goal was to identify characters whose relative timing of appearance would be consistent. As such, we ended up excluding several classes of juvenile characters that we identified as “heterochronic” in their appearance from larva-to-larva or batch-to-batch, notably, those outside of the rudiment proper (see Additional file 1: Figure S1). In the final staging scheme, we thus settled on characters that are defined by discrete morphological features (soft and hard structures) within the developing juvenile rudiment of *S. purpuratus* larvae, visible in live specimens either under differential interference contrast (DIC) or cross-polarized light. A detailed description of these stages is presented in the Results section and in Tables 1 and 2.

Our data on approximate numbers of days from fertilization to various stages (see third column in Tables 1 and 2) are compiled from our numerous fertilizations and rearings in different seasons and locations at 14ºC and under our various culturing conditions outlined above. We therefore believe that the results are robust with respect to population differences in different geographic locations, as well as water chemistry and other factors. We note that these timing data are truly approximations, as larvae in a given batch can vary substantially from these values, both in mean stage and in the variance among stages. For example, some batches of larvae, for reasons that we and others have not identified, undergo bouts of asexual larval budding [45,91,92], sometimes in a majority of the larvae within a culture vessel (data not shown). The resultant larval cultures are both delayed and more variable than larvae from a more typical rearing.

### Stage length experimental design

To determine with more precision the lengths of individual skeletogenic stages, we conducted staging observations on individual larvae in two of our rearing locations. The majority of our staging data derive from larvae that we cultured at UOG in June 2011. We also report on our temperature comparison experiment conducted in SEA in September 2011. Throughout the Methods and Results sections, we refer to our ‘Guelph’ or ‘Seattle’ experiments, respectively.

For the Guelph staging studies, we selected 48 larvae on day 28 that had visible rudiments, and mounted them individually on a microscope slide with raised cover glass using modeling clay, in order to immobilize but not damage the larvae. We then used cross-polarized and DIC optics to stage each larva (0 hour time point) according to our staging scheme (see Results and Tables 1 & 2), photographed it (see below), and gently transferred it to an individual well in a 24 well plate (Costar 3524) with 1.5 ml of MFASW and algae at the same concentrations as in their rearing conditions (see above). We maintained the well plates for 24 hours at 14°C, at which point we again mounted and photographed each larva as above, and assigned it to a stage (24 hour time point). We continued to culture 24 of these larvae for an additional 24 hours in their same wells, and photographed and staged them one final time (48 hour time point).

The Seattle protocol was similar to that described above with the following differences: 1) the experiment began on day 21, so the experimental larvae only covered skeletogenic Stages 0–6; 2) we assigned 48 larvae at random to either a 12°C or 16°C treatment to examine temperature effects on staging progression; 3) we staged each larva at 0, 24, 48 and 96 hours of the experiment; and 4) we fed larvae for the duration of the experiment at the level at which they were fed throughout development (see above), with a full water change at 48 hours.

### Stage length calculations

For both the Guelph and the Seattle datasets, we made the following three assumptions for the calculation of skeletogenic stage lengths (in hours) for each observed larva: 1) since larvae were observed at most once per day, larvae at each observation point were assumed to be at the mid time point of the observed stage; 2) if a larva advanced one or more stages during an observation time interval (i.e. 24 or 48 hours), that larva was assumed to have progressed through all intervening stages at a constant rate (calculated as the time interval divided by the stage differential); and 3) if a larva did not progress at all during a 48 hour observation interval, the total length of that stage for that larva could not be estimated, so we scored the length of that stage for such larvae as 72 hours. We consider this maximum length of 72 hours to be quite conservative, based on our observations on cohorts of larvae that indicate that none of our skeletogenic stages are of that duration at 14-16°C (data not shown).

For example, the length of stages for a hypothetical larva that began the experiment (0 hrs) at Stage 6, progressed to Stage 7 at 24 hours, and then Stage 9 at 48 hours would be computed as follows. Based on assumption 1: the larva was at the midpoint of Stage 6 at 0 hrs, of Stage 7 at 24 hours and Stage 9 at 48 hours. Sometime during the second 24 hours, the larva entered and exited Stage 8. Based on assumption 2: the lengths of Stage 7, 8 and 9 are assumed to be equal, and thus 12 hours each (time interval = 24 hours, stage differential = 2 stages; therefore 24/2 = 12 hr). This means that Stage 7 is assumed to have begun 18 hours into the experiment, Stage 8 at 30 hours, and Stage 9 at 42 hours). And, finally, Stage 6 – which was at its midpoint at 0 hrs (see assumption 1) – is 36 hours for this larva (it is assumed that Stage 6 for this larva began 18 hours before the onset of the experiment and finished when Stage 7 is assumed to have begun: namely, 18 hours after the start of the experiment).

We calculated means, standard errors of the mean and the 95% confidence intervals for the lengths of each stage at each temperature. Note that we could not estimate the length of Stage 10 in our study, since we calculated stage lengths based on progression into the subsequent stage, and there is no Stage 11 in our scheme.

In order to confirm that our live mounting technique of larvae did not affect the developmental progression of larvae, we setup a separate experiment with a total of 24 larvae in February 2014 at UOG. 12 larvae were randomly assigned to a mounting treatment. Specifically, we mounted these 12 larvae as described above, staged them and placed them back into individual wells as described above. The other 12 larvae were placed directly into wells without mounting. After 48 hours, all 24 larvae were staged, and we compared the distribution of stages in the two sets of larvae (manipulated vs. non-manipulated).

### Spine growth

Purple urchin larvae have two types of spines: 6-sided “adult” spines and 4-sided “juvenile” spines (see [39]) for review, and for a listing of alternative names in the literature for these spine types). The latter are so named as they are juvenile-specific, and are not found in urchin adults. Due to their greater numbers, ease of scoring and regular positions within the growing rudiment, we decided to focus exclusively on the adult spines for this particular analysis. Using the same dataset described above (Guelph experiment), we counted the number of cross hatches in the first five adult spines that we could observe in each larva that had reached Stage 8. We used these data to calculate mean and maximum number of cross hatches for each larva.

We calculated growth rates of the adult spines within the first and second 24 hour period of observations to get a rough estimate of spine growth in *S. purpuratus* larvae. For this analysis we counted the regular cross bars that occur along the length of the growing adult spines (see Figure 5A) within the rudiment during the final week or so of development before settlement; since these bars occur at regular intervals along the length of the growing spine (data not shown), the number of cross bars (=cross hatches) is a convenient way to estimate spine length. For each larva in the Guelph experiment, that was at or beyond Stage 8 (see Table 2), we counted the number of complete cross hatches (i.e. with no gap) in the first five adult spines that we identified while examining larvae at 0, 24 and 48 hours. We then calculated the mean (Avg) and maximum (Max) number of cross hatches for the five counted spines in each of these larvae, and used these values to compare mean rates of spine growth (for both Avg and Max) during the first and second 24 hour periods of observation.

Note that adult spines are hexagonal around the long axis of the spine, and thus have cross bars along all six edges of the hexagon (i.e., along all six faces of the spine), and that the number of cross hatches along each of these six faces is not always consistent within a spine (e.g., see Figure 5A). We counted the maximum number of cross hatches visible on any of the six faces. For example, in a spine where the six faces had 2, 2, 3, 2, 2 and 3 cross hatches, it would receive a score of 3 cross hatches. In another example, if only one complete cross hatch appears on as few as one face, that spine would receive a score of 1 cross hatch. We analyzed these data using a one tailed *t*-test for 24 and 48 h in order to assess whether the rate of addition of cross hatches was different during the first and second 24 hours of our experiment.

### Comparison to other staging schemes

In Tables 1 & 2, we present comparisons between our staging scheme and the staging schemes for *S. purpuratus* and 3 other echinoids, as presented by Smith et al. [37] and Chino et al. [38] respectively. For these comparisons, we examined the images, drawings and descriptions presented by these authors, and compared them to the new soft tissue and skeletogenic stages that we present here.

### Imaging

In Guelph, we imaged larvae on a Nikon Ti microscope equipped with a motorized stage. Images of larvae were taken at each time point and, if necessary, multiple views were captured of juvenile skeletons. In Seattle, we imaged only a select subset of larvae to illustrate certain stages, using a Nikon Coolpix 990 camera mounted on a Leitz Wetzlar Ortholux microscope. We captured additional images to illustrate some stages in Figure 1 using a Zeiss Axio Imager Z1 microscope at HMS. All three of these systems were equipped with DIC optics and cross-polarized light. All image are oriented with posterior to the left.

### Ethics statement

Research presented here does not require any approval and therefore complies with all necessary regulations.

## Competing interests

The authors declare that they have no competing interests.

## Authors’ contributions

AH and JAH carried out experiments at the University of Guelph and Hopkins Marine Station (Stanford University), JAH carried out additional experiments at the University of Washington (Seattle), at Friday Harbor Laboratory, and at Bodega Marine Laboratories. Experimental planning, analysis of data and writing of the article were joint efforts by AH and JAH. Both authors read and approved the final manuscipt.

## Supplementary Material

Additional file 1: Figure S1 Non-rudiment juvenile skeletal elements develop asynchronously with respect to skeletal elements inside the rudiment, and are therefore not included in our staging scheme. **(A-D)** Cross-polarized light images of living larvae: **(A, B)** Anal view (sensu [37]), therefore rudiment at right; **(C, D)** Abanal view (sensu [37]), therefore rudiment at left. **(A)** Stage 3 larva with a more fully developed genital plate 2 (the “madreporic plate”; white arrowhead) than the Stage 5 larva in **(B)**. White arrow in **(B)** points to an incomplete first tube foot ring, a feature identifying this larva as Stage 5. **(C)** Stage 6 larva with a more fully developed genital plate 5 (white arrowhead) than the Stage 8 larva in **(D)**. **(E, F)** Light micrographs of Stage 10 larvae (white arrow in each panel points to tube feet with complete second tube foot rings), compressed under cover glass to flatten all skeleton into a single focus plane; rudiment at left. These are composite images, as it took two images to visualize all of the skeleton in each larva. The respective adult spines in the two larvae have approximately the same numbers of cross hatches. But the right posterior juvenile spine (white arrowhead) in **(E)** is a pre-spine, with no cross hatches, whereas the corresponding juvenile spine in **(F)** has two cross hatches. Scale bars **A**: 200 μm; **B**: 150 μm; **C**: 280 μm; **D**: 240 μm; **E**, **F** - 90 μm. Click here for file
