# Supplementary figures and images for: A detailed staging scheme for late larval development in Strongylocentrotus purpuratus focused on readily-visible juvenile structures within the rudiment
Source: BMC Dev Biol. 2014 May 19;14:22. doi: 10.1186/1471-213X-14-22 (PMC4055376; doi:10.1186/1471-213X-14-22)

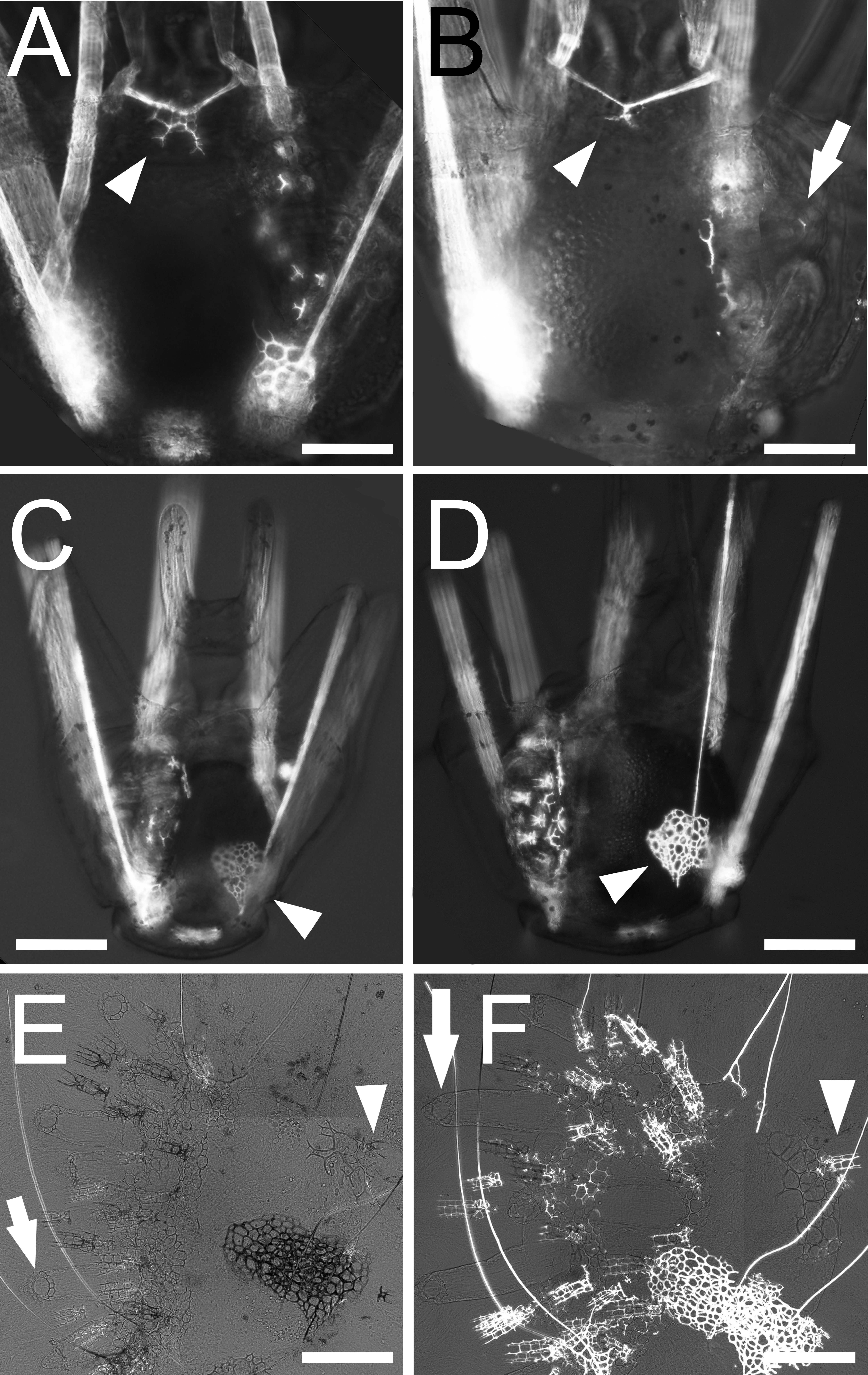

Supplement: Additional file 1: Figure S1 — Non-rudiment juvenile skeletal elements develop asynchronously with respect to skeletal elements inside the rudiment, and are therefore not included in our staging scheme. (A-D) Cross-polarized light images of living larvae: (A, B) Anal view (sensu [37]), therefore rudiment at right; (C, D) Abanal view (sensu [37]), therefore rudiment at left. (A) Stage 3 larva with a more fully developed genital plate 2 (the “madreporic plate”; white arrowhead) than the Stage 5 larva in (B). White arrow in (B) points to an incomplete first tube foot ring, a feature identifying this larva as Stage 5. (C) Stage 6 larva with a more fully developed genital plate 5 (white arrowhead) than the Stage 8 larva in (D). (E, F) Light micrographs of Stage 10 larvae (white arrow in each panel points to tube feet with complete second tube foot rings), compressed under cover glass to flatten all skeleton into a single focus plane; rudiment at left. These are composite images, as it took two images to visualize all of the skeleton in each larva. The respective adult spines in the two larvae have approximately the same numbers of cross hatches. But the right posterior juvenile spine (white arrowhead) in (E) is a pre-spine, with no cross hatches, whereas the corresponding juvenile spine in (F) has two cross hatches. Scale bars A: 200 μm; B: 150 μm; C: 280 μm; D: 240 μm; E, F - 90 μm. [file 1471-213X-14-22-S1.jpeg]
